# Elucidating the Macroeconomic Determinants of Undernourishment in South Asian Countries: Building the Framework for Action

**DOI:** 10.3389/fpubh.2021.696789

**Published:** 2021-08-12

**Authors:** Noshaba Aziz, Jun He, Ali Raza, Hongguang Sui, Wang Yue

**Affiliations:** ^1^College of Economics and Management, Nanjing Agricultural University, Nanjing, China; ^2^OYAGSB, Universiti Utara Malaysia, Sintok, Malaysia; ^3^School of Economics, Shandong University, Jinan, China

**Keywords:** undernourishment, economic growth, food price index, governance, South Asia

## Abstract

Undernourishment is a big challenge for humanity across the world. Considering the significance of reducing undernourishment, the current study focuses on exploring the macroeconomic determinants of undernourishment in the South Asian panel. The study employed econometric models that are more robust to underpin cross-sectional dependency and heterogeneity in a panel data set. The overall findings reveal that an increase in food production increases undernourishment and infer that food availability at the national level is insufficient to reduce undernourishment unless poor people also had economic and physical access to food. In the case of economic growth and governance, the results are negatively significant in some countries. The results infer that GDP and quality of governance are nuanced in declining the rate of undernourishment in some countries, while in other countries where the results are found insignificant, the government should seek other interventions to curtail the prevalence of undernourishment. Unexpectedly, an increase in food prices lessens the undernourishment in developing countries that reflect that food prices might transform the dietary patterns of poor people from nutrient-rich foods to nutrient-poor staples, thus lead to undernourishment reduction but trigger overweight and obesity alongside. In conclusion, the results depict that policymakers should devise strategies keeping in view fundamental aspects of the country to reduce undernourishment.

## Introduction

Undernourishment is one of the triple burdens of diseases that affect inhabitants globally (1). It is regarded as a situation in which the dietary energy consumption of people required to sustain healthy lives is unremittingly below the minimum energy requirement (2). Generally, undernourishment is pondered as a primary factor leading to diverse health consequences, such as weak immune and cognitive systems and poor health growth. Previous literature has also shown that undernourishment unfavorably influences the physical and mental health of humans (3, 4). Many global commitments and efforts have been driven to reduce undernourishment; still, about 2 billion people worldwide could not access safe, nutritive, and ample food in 2019 (5). The world is far away from accomplishing Sustainable Development Goal 2 of ending world hunger by 2030, given the fact that ~840 million people still adhere to face serious food insecurity by the end of the decade (5). Feeding people living in resource-limited economies is a grave concern that poses the inability of policymakers. Therefore, identifying determinants of undernourishment at a macro level is of great importance for policymakers, which entails a strenuous and wise approach to addressing this urgent humanitarian crisis.

Globally, the undernourishment prevalence has been stabilized, but the total figure of the undernourished population is rampantly increasing (5). Currently, the COVID-19 outbreak further worsened the situation by putting negative and ripple effects on every aspect of the lives of peoples, i.e., pandemic-induced economic shocks, such as travel restrictions, job, and income loss, have disrupted the livelihoods and food supply chains globally (6–8). According to the recent report on State of Food Security and Nutrition in the World (2020), it is found that additional 132 million people faced undernourishment by the end of 2020 due to the pandemic. The COVID-19 deteriorated the dietary status of the unprivileged people by affecting their socioeconomic conditions (5). This report further warned that COVID-19 is universally jeopardizing the hunger situation (9).

Worldwide, to increase the living standard of people, the United Nations has established 17 development strategies commonly known as “sustainable development goals” (SDGs) that encompass multiple developmental programs for several aspects of life and are expected to be attained by 2030 (10). But, unfortunately, the COVID-19 has posed an austere threat to these developmental programs. And according to the SDGs of zero hunger, food insecurity and hunger are still a universal challenge despite all potential efforts (11–15). Beholding the near future, it is pertinent to say that undernourishment is a challenging phenomenon worldwide, and, if the undernourished people keep on growing with the same momentum globally as in 2019, it will be difficult to accomplish an SDG of zero hunger (5). Globally, most food-insecure people are inhabited in Asia (5). Although progress has been made toward minimizing food insecurity globally, Asia also falls under the category of having food insecurity and prevalence of undernourishment, following Africa and Latin America and Caribbean (5).

Among Asia, food insecurity is a grave concern in the South Asian region. Although the potential of agriculture in the region is favorable, there are still other challenges, such as low output, population pressures, and an increase in pesticides risk (16). Among South Asian countries, Table 1 depicts that India, responsible for 70% of the hunger of the region, has shown a reduction in the prevalence of undernourishment^1^ by 36% regardless of the increased population by 48% in the past quarter of a century (17). Although the number of undernourished people is reduced in the other parts of the regions, the condition in Afghanistan, Pakistan, and Bangladesh is still worst and challenging (17). The number of undernourished people in Afghanistan raised from 3.8 to 10.6 million during the years 1990 to 2018, whereas, during the same period, the number increased from 28.7 to 40 million in Pakistan. It is projected that if south Asian countries keep on making progress based on the same trends, they cannot attain the target of zero hunger by 2030 (5).

**Table 1:** Undernourishment trends in South Asian countries.

**Region**	**1990–1992**		**2016–2018**	
	**No. of undernourished people (million)**	**Undernourishment prevalence (%)**	**No. of undernourished people (million)**	**Undernourishment prevalence (%)**
South Asia	291.2	23.9	277.7	14.9
Afghanistan	3.8	29.5	10.6	29.8
Bangladesh	36	32.8	24.2	14.7
India	210.1	23.7	194.4	14.5
Maldives	<0.1	12.2	<0.1	10.3
Nepal	4.2	22.8	2.5	8.7
Pakistan	28.7	25.1	40	20.3
Sri Lanka	5.4	9	1.9	22

*Source: FAO (17)*.

Given the widespread food insecurity and undernourishment among children in the South Asian region, the COVID-19 pandemic may reverse the progressive trends of poverty mitigation with disastrous and far-reaching significances. In South Asian countries, the current pandemic further sparked fear of an impending economic crisis and recession and even disturbed the ability of people to carry out their actions. Besides pandemics, several other factors may cause food insecurity and undernourishment, for instance, sociocultural, political and economic factors, such as conflict, fluctuations in commodities prices, and natural disaster shocks may also exacerbate food insecurity and undernourishment (18, 19).

In the previous literature, many studies focused on measuring food insecurity at the microlevel concerning the individuals and ability of households to attain food security (20–29). But, Pinstrup-Andersen (30) and Ecker and Breisinger (31) argued that, although household-level food security is essential, it does not measure the required conditions to maintain adequate nutrition. In 2011, the United Nations recognized that the nutritional development goal must be attained at the national level beyond the household level. At the macro level, factors that measure food security include economic growth (32, 33), public expenditure, and investment in socioeconomic sectors, governance, and institutional quality sectors (31, 34). These factors may support the increase in incomes, assets, and services of the households and ultimately secure the nourishment and food security of the households (31).

Moreover, the nutritional aspect in food security is regarded as a proximate determinant for improving cognitive and physical growth of humans and leads to enhance labor productivity and efficiency (35) and the overall economic development of the region (36, 37). Unquestionably, the cumulative impact of the factors escalating the severity of undernutrition in South Asian countries is thought-provoking and requires a considerable commitment and solemnity by the governments to address this issue. Hence, the current study attempts to analyze the nutritional status by assessing macroeconomic data in South Asia from 2000 to 2019. The undernourishment is measured by using the prevalence of undernourishment (POU) (see text footnote 1) variable, and the macroeconomic determinants include the average value of food production, economic growth, and, most importantly, governance, as well as prices of goods, which has not received much attention in the previous years.

Given the limited literature on this issue, especially in South Asian countries, the current study intends to fill the research gap by offering the following contributions. Firstly, the significant flaw in the existing panel studies is cross-sectional dependency and heterogeneity issues, and econometric approaches that do not consider heterogeneity and cross-sectional dependency across the panel may lead to biased results. So, the current study applied fully modified ordinary least square (FMOLS) and dynamic ordinary least square (DOLS) to gain robust and unbiased results, and, for this, the study initially applied the cross-sectional dependency (CD) test of Pesaran (38). Secondly, it is debated that conventional unit root tests provide rigorous results when data series are cross-sectionally dependent; so, this study applied cross-sectional IPS (CIPS) panel unit root test developed by Pesaran (39), which assumes cross-section dependency. Thirdly, this study additionally employed the bootstrap panel cointegration test of Westerlund (40) and the test statistics of Kao (41) to elaborate the cointegration. And, in the end, FMOLS and DOLS tests are applied to get vigorous results in the long run. Many recent studies have also used this phenomenon [see (42, 43)].

The remaining paper is structured as follows. The possible existing studies on the topic focused in the present study are reviewed in section Literature Review. Data sources and empirical estimations are offered in section Methodology. Based on estimations, the results are provided in section Empirical Results, and a conclusion with potential policy implications is revealed in section Conclusions and Policy Recommendations.

## Literature Review

This section attempts to divulge the earlier efforts of the researchers on the concerned variables in different strands. The first strand encompasses studies exploring the nexus between the average value of food production and undernourishment. The following strands encompass the nexus of undernourishment with food prices, economic growth, and governance.

The main factor responsible for food security, particularly for predictable population growth under increased climate variability (44, 45), is food production (46, 47). The agricultural production of food is imperative to source nutritious foods, reduce the food prices, and raise the incomes, mainly for the deprived smallholder farmers in low- and high-income countries. Recently, Mughal and Charlotte (48) have described agricultural production as a front door to reduce undernourishment prevalence. Agricultural growth is an alternative that abolishes the susceptibility of starvation by enhancing the output (49). Agricultural output and childhood underweight are positively associated, which is also proven by Balk et al. (50). The study of Godecke et al. (51) proves that both larger food supplies and diversity help reduce chronic hunger.

Moving toward economic growth, some studies report that economic development is a precondition to enhance the nutritional status of people in developing countries (52, 53). Soriano and Alberto (54) paper shows that it takes 2 years for income growth to improve the prevalence of undernourishment. The research of other scholars also evidences that improvement in the undernourishment prevalence leads to economic growth (55–58). Heltberg (59) conducted a study that shows that economic growth has little but substantial effects on child malnutrition. Haddad et al. (60) study shows that national income accompanied by unique feeding services may help lessen child malnutrition. The study of Subramanyam et al. (61) and Kumar (62) in India concludes that economic growth declines undernourishment of the children. Economic growth, which is fundamental to reduce undernutrition, is also proven by Ecker et al. (63). But the study of Warr (64) argues that economic growth does not help countries decrease undernourishment. The outcome is only found significant for Asia, but as far as Africa and Latin America are concerned, the results are not found significant.

Surprisingly, some recent studies have shown that developed countries are also experiencing malnutrition (65) and highlight developing strategies and policies to reduce nutritional challenges (66, 67). Few scholars argue that food security is not as reactive to economic growth as other human development measures, such as poverty (61, 68). Income growth is an essential but not enough factor required to combat undernourishment, and the reason could be the unequal distribution of income among the masses (69). The study of Subramanyam et al. (61) unveils some factors accountable for the frail connotation between undernourishment and economic growth. For instance, firstly, the impact of the economic growth on nutrition and food security of an individual is based on the proportion of poor people who see their income grow as there is a substantial disparity in the share of poor people in the aggregate economic growth (70, 71). Secondly, in some countries, other interventions of the government and relatively lower *per capita* income help countries improve their food situation (32). Thirdly, economic growth needs to be coupled with increased income and wealth of households, along with education, clean water, and health care services (52, 72). In this scenario, it is notable that income growth alone may not help countries improve the nourishment of the people.

Meanwhile, another variable such as governance is regarded as a necessary factor to promote an effective environment for improving regional food and nutrition security and economic growth. Governance acts substantially on improving food security (34, 73, 74). An enhanced democratic government helps decrease the undernutrition of children (75, 76). A recent study by Ogunniyi et al. (77) shows that remittances and composite quality control index improve the average production of food in Sub-Saharan Africa. Several other studies have proved that the capacity of policy interventions maintains the food demand and supply positively (78, 79). Food insecurity of any country is directly related to unstructured institutes or the inability of the state to impose policy interventions to guarantee legal rights to its people (80, 81). In Sub-Saharan African countries, Bello- Schünemann and Moyer (82) state that instability and unrest of political institutions raise an intense conflict that threatens the whole regional well-being by influencing their nutrition. The meager governance system weakens both the performance and policies of institutions (83). Besides, the availability of assets that reflect the degree to which people are food secure also depends on the efficacy of the public institution (84). The more the resources manage efficiently by the institutions, the more the people will be secure. Consequently, to devise and implement approaches necessary to boost food and nutritional security, the role of government should be strengthened (74, 85).

Apart from the above-discussed variables, undernourishment also depends on the prices of food compared with other commodities. Generally, higher food prices lead people toward the higher menace of undernourishment. Globally, in almost all regions, healthy diets are too expensive for deprived people. In the existing literature, there is the bulk of studies that have explored the price elasticities of food demand (86–91), and their findings show that different food prices influence the micronutrients consumption differently. In another study, Nandy et al. (92) portray that higher food prices lead to higher prevalence and numerous anthropometric failures in children in Nigeria. The study of Anríquez et al. (93) also exhibits the same findings and states that undernourishment increases with increased food prices. Many other studies show that extreme seasonal price fluctuation results in famines (94–98).

Keeping in view the above discussion, it would be better to say that the analysis of macroeconomic determinants could be used as a tool for policy design to halt undernourishment, particularly in the perspective of South Asian countries. To our knowledge, there is no study available in the previous literature that examines the impact of an average value of food production, economic growth, food price index, and quality of governance on undernourishment in South Asian countries. To fill the gap, the current study is attempting to contribute to the prevailing literature.

## Methodology

### Data Sources

The core aim of the present study is to elucidate the macroeconomic determinants of undernourishment in South Asian countries. To meet the objective, the current study uses the prevalence of undernourishment, the average value of food production, food price index, economic growth, and governance. The study includes Afghanistan, Bangladesh, Nepal, Iran, India, Pakistan, and Sri Lanka, given that the data of POU for Maldives and Bhutan are not available. All data of variables are collected from World Development Indicators data bank, excluding governance collected from World Bank governance indicators, covering 2000 to 2019. Table 2 summarizes the data sources and descriptions of variables.

**Table 2:** Explanation of variables.

**Abbreviations**	**Variables**	**Measurements**	**Data sources**
POU	Prevalence of undernourishment	3-years averages	WDI
AVP	Agricultural food production^a^	2004–2006 U.S. per capita output	WDI
GDP	Economic growth	Current US dollars	WDI
CGI	Composite governance index^b^	An index made by taking the average of all indicators	WGI
FPI	Food price index^c^	Price Index	WDI

a *The agricultural production portrays the relative level of the aggregate volume of agricultural production for each year in contrast to the base period 2004–2006. They are based on the sum of price-weighted quantities of different agricultural commodities produced after deductions of quantities used as seed and feed weighted in a similar manner. The resulting aggregate, therefore, signifies disposable production for any use except as seed and feed (https://www.indexmundi.com/facts/indicators/AG.PRD.FOOD.XD)*.

b *Composite governance index (CGI) is measured by averaging its core indicators, such as corruption control, the stability of politics, effectiveness of government, the rule of law, quality of regulation, and voice and accountability domains*.

c *The food price index is calculated as the trade-weighted average of the prices of food commodities spanning the key agricultural markets for cereals, vegetable oils, sugar, meat, and dairy products. While these commodities represent about 40 percent of gross agricultural food commodity trade (FAOSTAT), they are chosen for their high and strategic importance in global food security and trade. Prices are combined in the various sectors, using trade weights calculated from average export values over a chosen 3-year-based period, when the trade weights appear most stable relative to their trend values. A 3-year period is chosen to minimize the impact of variation in both internationally traded prices and quantities (www.fao.org/fileadmin/templates/worldfood/Reports_and_docs/FO-Expanded-SF.pd)*.

### Descriptive Statistics

In Table 3, the current study primarily portrays the descriptive statistics of the variables. The results reveal that maximum and minimum values of POU for the sample countries are about 47.8 and 4.5, respectively. The food production in the sampled countries has a mean value of 172.3389, and the standard deviation is about 67.43421, with the largest value of 339 and the smallest value of 89.95. The mean value of GDP is nearly 1608.627 US dollars, and its standard deviation is about 1683.544 US dollars. The composite governance index has a mean value of −0.827149 with a standard deviation of 0.459894. The mean value of the food price index is 8.244, with a 6.4 standard deviation value.

**Table 3:** Descriptive statistics.

	**POU**	**AVP**	**GDP**	**CGI**	**FPI**
Mean	15.178	172.339	1608.6	−0.827	8.244
Median	14.350	163.000	969.91	−0.863	6.956
Maximum	47.800	339.000	7927.8	−0.069	39.90
Minimum	4.500	89.950	158.63	−1.981	−6.811
Std. dev.	8.525	67.434	1683.5	0.460	6.479
Observations	140	140	140	140	140

*Source: Author(s) estimations*.

### Model Specification

The current study aims to explore the macroeconomic determinants of undernourishment in a panel of South Asian countries. Under this framework, the primary step is to check each stationarity of variables before moving to the cointegration tests. Stationarity is essential for the panel and time-series data because non-stationary data in empirical estimations lead to spurious regression results (99). Thus, Im et al. (100) unit root test is employed to check the stationarity. In panel data, it is argued that conventional unit-root tests may not perform well and possess the likelihood of cross-sectional dependency (CD) in the data series. So, second-order generation tests, such as cross dependence (CD) and augmented cross-sectional IPS (CIPS), are additionally applied, as these tests are performed under the notion of cross-sectional dependency and heterogeneity (39), which is likely to remain unnoticed by the first-order generation test of Im et al. (100, 101).

Once the order of integration is detected, the subsequent step is to check the cointegration association of variables. Cointegration tells us the presence of the long-run association among the studied variables. From unit root results, it is confirmed that the variables are integrated, and the panel bootstrapped cointegration test is now robust to apply (40). The current study employs Westerlund (40) bootstrap panel cointegration test. This method proposes more consistent values by reducing the cross-sections “distortional effects.” Test statistics of Kao (41) are also used to elaborate on the cointegration. The test statistics of Kao is used within the framework of the ADF approach. The study further employs FMOLS and DOLS; the “FMOLS is a non-parametric method to cope with corrections for serial correlation, while DOLS is a parametric method where lagged first-differenced terms are evidently assessed. By using DOLS, the residuals are amplified with lags, lead, and contemporaneous values of the regressors.”

As the current research aims to elucidate the determinants of the prevalence of undernourishment in desired South Asian countries, therefore, the following model is structured to analyze the determinants s empirically:

(1) $$\begin{array}{lllll} POU_{i,t} & = & \alpha_{0}+\beta_{1}POU_{i,t-1}+\beta_{2}AVP_{i,t}+\beta_{3}GDP_{i,t}{+\beta }_{4}CGI_{i,t} & + & \beta_{5}FPI_{i,t}+\mu_{i,t} \end{array}$$

where *POU, AVA, GDP, CGI*, and *FPI* signify the prevalence of undernourishment, average value of food production, food price index, economic growth, and composite governance index, respectively. In this model, the prevalence of undernourishment is the dependent variable, and βk (*k* = 1, 2, 3, 4, and 5) are the coefficients of the lag of prevalence of undernourishment, average value of food production, food price index, economic growth, and composite governance, and μ_*i,t*_ shows the error term.

Apart from FMOLS and DOLS, the study further employed the Granger causality test promoted by Dumitrescu and Hurlin (102) to inspect causal relationship of variables, and it is reliable in panel cases when the error terms are cross-sectionally dependent.

## Empirical Results

### Unit Root Tests

As a preliminary step, the stationarity properties of the variables are examined, using the conventional unit root tests introduced by Im et al. (100). The unit root test results with individual intercept (C) and individual intercept with trend (C and T) term are stated in Table 4. The results depict that AVP and GDP contain unit root at the level but become stationary at the first level.

**Table 4:** Stationary analysis results.

**Variable**	**I'm Pesaran and Shin**			
	**I(0)**		**I(1)**	
	**C**	**C and T**	**C**	**C and T**
POU	−1.6510^**^	−8.03518^***^	−4.30267^***^	−4.06693^***^
AVP	−0.1694	0.93898	−1.90064^**^	−2.0114^***^
GDP	2.936	1.89565	−2.37356^***^	−2.37356^***^
CGI	−2.520^***^	−1.78373^**^	−4.29050^***^	−2.75328^***^
FPI	−2.467^***^	−0.57278	−7.05133^***^	−6.45034^***^

***, **, and * *indicate levels of significance at 1, 5, and 10%, respectively*.

Furthermore, the CD unit root test results also support the alternative hypothesis of cross-sectional dependency for all variables at the 1% significance level, indicating the presence of cross-sectional dependency among sampled countries (see Table 5). The CIPS unit root test results also support the alternative hypothesis for all variables at the level and first difference and signify the non-stationary behavior of all variables.

**Table 5:** Cross-sectional dependence and CIPS unit root test results.

**Variables**	**CD test**	***p*-value**	**CIPS test**	
			**Level**	**1st difference**
POU	13.39086	0.000	−4.49^***^	−2.22^***^
AVP	7.568020	0.000	−1.41^*^	−1.81^**^
GDP	19.03166	0.000	2.36^*^	−2.32^***^
CGI	5.443783	0.000	−3.93^***^	−4.628^***^
FPI	7.422865	0.000	−2.38^***^	−2.99^***^

***, **, and * *Signify the significance level at 1, 5, and 10%, respectively*.

### Panel Cointegration Test

After confirming the stationarity of variables, the current study also employs panel cointegration tests of Kao (103) and Westerlund (40) bootstrapped cointegration tests to measure the long-run association. Under Westerlunds (40) bootstrapped cointegration tests, the results in Table 6 accept the alternative hypothesis by rejecting the null hypothesis. Thus, the long-run cointegration of desired variables in the nourishment model is approved by this test.

**Table 6:** Westerlund (40) bootstrap panel cointegration test.

**Model 1 POU** **=** **f (AVP+** **GDP+CGI+FPI)**				
**Statistics**	**Values**	***Z*-values**	***P*-values**	**Robust *P*-values**
Gt	−4.112^***^	−5.114	0.000	0.000
Ga	−16.524^***^	−4.983	0.000	0.000
Pt	−10.785^***^	−6.859	0.000	0.000
Pa	−16.632^***^	−5.478	0.000	0.000

*Null hypothesis, i.e., no cointegration among model variables. The test is performed under 500 bootstraps replications*.

*Source: Estimation of Authors*.

***, **, and * *indicate levels of significance at 1, 5, and 10%, respectively*.

And, according to Kao (103), the cointegration between variables is also confirmed in the long run (see Table 7) as the ADF value is statistically significant.

**Table 7:** Panel cointegration test (POU) of Kao.

	***t-*value**
ADF	−3.157^***^
Residual variance	1.096
HAC variable	2.761

*** *Signifies a significance level at 1%*.

### Panel Estimation Results

#### FMOLS and DOLS Results for the Whole Panel

Tables 8, 9 show the FMOLS and DOLS results both for the whole panel and individual countries. The results in the case of AVP depict negative and significant effects on the prevalence of undernourishment in the FMOLS estimator, but, in DOLS, the results are unexpectedly positive and unveil that a 1% increase in AVP positively influences undernourishment by 58%. Unlike the previous studies, the results are quite surprising for DOLS estimators. The study used the average value of food production and is deliberated as a path to ensure food security (104). It is assumed that higher production of food is linked with a decline in undernourishment. But, in our case, the results are not surprising because it has been acknowledged for decades that food availability at the national level is not enough to ensure food security to individuals, but that access to food should also be ensured (105). The results infer that only increasing the global food production and ensuring that all people everywhere have enough food to eat is not enough. The sufficiency of food supply at the national level is not enough to assure food security at the regional level unless poor people also had economic and physical access to that food (106, 107). The result aligns with the findings of Howeida and Zeinab (108), who also found that the food production index is not conducive to a decline in the prevalence of undernourishment.

**Table 8:** FMOLS and DOLS long run results of POU.

	**FMOLS**			**DOLS**		
**Variables**	**Coefficient**	**Std. error**	***t*-stat**	**Coefficient**	**Std. error**	***T*-stat**
AVP	−0.160^***^	0.023	−6.974	0.588^***^	1.13E-09	5.20E+08
GDP	−0.005^***^	0.001	−6.763	−0.009^***^	6.92E-12	−1.28E+09
CGI	−3.142^***^	0.927	−3.391	−43.134^***^	1.17E-07	−3.68E+08
FPI	−0.107^***^	0.026	−4.137	−2.599^***^	5.35E-09	−4.86E+08

*Source: Estimations of authors*.

***, **, and * *indicate levels of significance at 1, 5, and 10%, respectively*.

**Table 9:** Cross country analysis of POU.

	**Afghanistan**	**Bangladesh**	**India**	**Iran**	**Nepal**	**Pakistan**	**Sri-Lanka**
	**FMOLS**						
**Variables**	**Coefficient**	**Coefficient**	**Coefficient**	**Coefficient**	**Coefficient**	**Coefficient**	**Coefficient**
AVP	−0.092	0.010	−0.258^***^	0.008	−0.112^***^	−0.349^***^	−0.327^***^
GDP	−0.027^***^	−0.002	0.004^***^	−0.000^***^	−0.010^***^	−0.003	0.000
CGI	−8.296	2.799	−13.115^***^	−2.078^***^	9.809^***^	−10.325^***^	−0.787
FPI	−0.239^**^	−0.076^***^	−0.174^***^	−0.009	−0.298^***^	0.082	−0.029
C	39.878^**^	17.268^***^	54.008^***^	1.719	48.156^***^	72.437^***^	47.425^***^
	**DOLS**						
AVP	−0.196	0.002	−0.227^***^	0.008	−0.116^**^	−0.333^***^	−0.329^***^
GDP	−0.033^***^	−0.002^**^	0.003^**^	−0.000^**^	−0.010^**^	−0.004	0.000
CGI	−8.429	3.204	−12.975^***^	−1.659	9.704^***^	−10.402^**^	−0.575
FPI	−0.219	−0.077	−0.153^*^	−0.009	−0.295^**^	0.045	−0.014
C	53.435	18.511^***^	49.548^***^	1.776	48.848^***^	70.075^***^	47.579^***^

*Source: Estimations of authors*.

***, **, and * *indicate levels of significance at 1, 5, and 10%, respectively*.

Moreover, the GDP and CGI results are expectedly significant and robust, negatively affecting undernourishment prevalence. Economic growth is broadly viewed as an essential and appropriate condition for improving the health of people. The results in the case of GDP depict that a 1% increase in GDP negatively influences the prevalence of undernourishment by 0.005% in the case of FMOLS and 0.0008% in DOLS. It deduces that the number of nourished populations can be increased with the increase in an income share. Moreover, lifestyle and dietary preferences can also be improved with increased income (109, 110). Many former studies have presented the same finding and reveal that increase in economic growth will lead to an increase in average income, which, in turn, will improve the access and consumption of goods and services that will ultimately lead to improving the nutritional and health status of the people (4, 60, 111, 112). The GDP can also increase the accessibility of households toward agricultural inputs and practices, such as organic fertilizer, better-quality seeds, and nutrient-dense food. The results correspond well with previous studies such as those of Subramanyam et al. (61), Kumar (62), Smith and Haddad (52), Summers and Pritchett (113), and Ravallion (53), who also evidenced that economic growth is a precondition to improve the nutritional status of the people living in developing countries. Given that nutritious food is a basic human right, economic growth can reduce undernourished people in South Asian countries.

In the case of governance, the result is found negatively significant and depicts that a 1% rise in governance negatively influences the prevalence of undernourishment by 3.14% in the case of FMOLs and 43% in DOLS. The results are expected and infer that the extent of food security in societies relies on the performance of the governance and the effectiveness of institutions. Globally, it is revealed that good performance of all factors significantly addresses food insecurity issues as they protect human rights and their access to economic resources. This result corresponds well with Mehta and Jha (114) and Dube and Phiri (83), who evidenced that poor governance lessens institutional performance and policy outcomes. Other previous studies also affirmed that poor management unfavorably influences the nutritional and food security of the population over the years (75, 82, 84, 115–117). In developing countries, it stimulates economic growth, which is anticipated to increase food supply (60, 118). Nasreddine et al. (119) also looked over the nutritional situation of the Eastern Mediterranean region and found the consensus of undernutrition and overnutrition in most areas associated with numerous economies.

The results in the case of food prices are quite shocking. Generally, it is believed that higher food prices lead to higher undernourishment. But, in our case, the results are quite the opposite and expose that an upsurge in prices reduces undernourishment as the FPI results are significantly negative by 10% in the case of FMOLS and 2.5% DOLS. It is not surprising that higher food price commodities ensure good and nutritious food for affordable people, but the reduction in undernourishment in the case of poor countries is quite surprising. It is noteworthy that there are two groups of people, i.e., sellers and buyers, in the market, so it is believed that price hikes may influence them differently. For instance, for net sellers of food products, increasing food prices can raise their real incomes. As a consequence, they can afford nutritious and expensive food (120). On the other side, upward change in prices affect net-buyer households capacity to afford nutritious food. In South Asian countries, most households are net buyers of food, and higher food prices make them more vulnerable and susceptible to the worst form of nourishment. It is also expected that the price hikes may change their dietary patterns and lead them to switch from nutrient-rich foods consumption, such as meats, vegetables, and other proteins, to nutrient-poor staple food, such as wheat and rice. In this case, higher food prices may reduce undernourishment but increase overweight and obesity alongside. Kochhar (121) also found that many countries in developing Asia were facing the double burden of fighting both under- and overnutrition and elaborated that social safety nets policies, including food transfer programs in South Asian countries, are helping poor people to fight against food price hikes and afford at least basic staple food. Swinnen (122) and Green et al. (98) also exhibited the same phenomenon and stated that higher prices of food adversely impact the nutritional status of the households, particularly deprived people, as falling in their buying power compels them to purchase inexpensive and less nutritious but cheap dense energy food. Rendering the study of Dizon et al. (123), the verdicts propose that, to reduce the undernourishment, the system requires focusing on securing access of all people to a balanced diet, coupling with crucial nutrients desired for an active and healthy life. Undernourishment can only be improved when individuals consume a healthy diet rather than simply having access to food required to fill their stomachs.

#### FMOLS and DOLS Results of Individual Countries

The cross-country analysis results provide notably contradictory but interesting findings among countries within the panel. Unlike the results of the whole panel, the results of FMOLS and DOLS reveal that an increase in agricultural food production decreases the undernourishment only in India, Pakistan, Nepal, and Sri-Lanka by 25, 11, 34, and 32%, respectively in the case of FMOLS, and 22, 11, 33, and 32%, respectively in the case of DOLS. The coefficients are close enough in both estimators, while, in other countries, the results are insignificant. The results of these countries infer that the mechanization of farming has improved over time, predominantly in terms of good quality seeds and tractor use, which is linked with increased yields of crops and, in turn, reduction of undernourishment in these countries. Likewise, the results of GDP are found negatively significant only in Afghanistan, Nepal, and Iran. Unexpectedly, the results of India are positively significant by 0.004% in the case of FMOLS and 0.003% in DOLS, which infers that an increase in GDP cannot help the country lessen the undernourishment. Additionally, the pandemic situation has rampantly jeopardized the health crisis and the economic fallout in India that further worsened the undernourishment situation of the country. So, in this instance, it is pertinent to say that economic growth is insufficient to eliminate undernourishment in India, and India needs other interventions to tackle undernourishment. The results align with the recent scholars who also witnessed the same findings (65–69).

In the case of governance, the results are only found significant in India, Iran, and Pakistan, while the results are insignificant for other countries. Shockingly, the results for Nepal are found positively significant. The results reveal that, although Nepal has been solidifying its governmental structures and institutions, the political tensions still run high (124). The political instability, poor governance, and conflicts among the political parties halt economic growth and ultimately lead to poor economic growth and undernourishment. This outcome corresponds well with the study of Bain et al. (125), who also stated that the higher corruption level adversely aggravates the malnutrition burden and its ensuing outcomes in the developing countries. So, Nepal is still lagging as compared with other South Asian countries. Rendering Rodrik et al. (34), it is pertinent to say that governance and food security can be both detrimental and beneficial; for example, it is believed that a nation with sufficient food security must be under stable control of government institutions. In contrast, food-insecure people must be under a country having a frail governance system.

Furthermore, the higher food prices reduce the undernourishment in Afghanistan, India, Bangladesh, and Nepal in FMOLS by 23, 0.07, 17, and 29%, and Nepal, again, by 29% in case of DOLS. The rationale is that social safety net programs in these countries enable them to have access to food. As a result, higher food prices do not affect them. Another case is the dietary transformation from nutrient-rich food to cheap energy-dense food that may lead to overweight and obesity (as discussed in the previous section).

### Heterogeneous Panel Causality Test

The results of panel causality are represented in Table 10. The results of the POU show that the bidirectional causality is presented between AVP and POU and between GDP and POU, indicating that both variables, i.e., the average production of food and *per capita* income, cause prevalence undernourishment, and prevalence of undernourishment also affects both variables in turn. In the case of the composite governance index, the unidirectional causality is present; for instance, POU causes governance, but the governance does not affect POU. The causality in the case of food prices is not present.

**Table 10:** Results of heterogeneous panel causality test.

**Null hypothesis**	**Statistics**	**Probability**
AVP does not homogeneously cause POU	3.792	0.000
POU does not homogeneously cause AVP	4.536	0.000
GDP does not homogeneously cause POU	4.606	0.000
POU does not homogeneously cause GDP	2.031	0.042
CGI does not homogeneously cause POU	0.693	0.489
POU does not homogeneously cause CGI	3.783	0.000
FPI does not homogeneously cause POU	−0.230	0.818
POU does not homogeneously cause FPI	0.966	0.334

*Source: Estimation of authors*.

## Conclusions and Policy Recommendations

The present research is conducted to spot the determinants of undernutrition and suggests possible implications for preventing and controlling this issue. Although South Asian countries are experiencing significant and fast economic growth, poverty reduction, food availability (126), and undernutrition are still highly prevalent in these countries.

The overall findings expose interesting findings, i.e., the increase in AVP increases POU in the whole panel data, but cross-country analysis showed that AVP reduces the undernourishment in India, Pakistan, Nepal, and Sri Lanka. Likewise, an increase in FPI increases the POU in the whole panel in the long run, but cross-country analysis shows that increased FPI reduces the undernourishment in Afghanistan, India, Bangladesh, and Nepal. In general, the results portray that safety net programs in developing countries enable their access to food in economic shock. Interestingly, in the case of India GDP is positively associated with undernourishment, it infers that India needs to invest more in peoples' food security to lessen undernourishment. In the whole panel, the results of governance reveal the negative association with POU, but, interestingly, only the governments of India, Iran, and Pakistan are showing favorable consequences to curb undernourishment. It reflects that the efficiency of institutions in these countries is imperative for economic activities, as they dictate the amount and efficacy of all investments in the market. In other countries, the results are insignificant and reflect the point that poor governance in the South Asian countries halts the economic growth and ultimately leads to undernourishment.

The overall findings revealed in the long run in the panel of south Asian countries that even if the average value of production and food prices falters, higher economic growth and good governance play a vital role in improving nutrition and food security in the long run in countries where the sound institutional environment manages the resources efficiently. Therefore, policymakers should be aware of a realistic approach to resolve the food crisis politically and economically. Undeniably, the attainment of undernourishment prevention necessitates the execution of suitable institutional plans and subsequent reinforcement of actions. In national food production, regional support should be provided to resolve food shortages at the regional level through joint actions. In the case of price hikes, the policy implication is that food safety nets supplement government approaches to improve the purchasing power and affordability of the most vulnerable populations toward healthy diets. Poor households should be encouraged to raise agricultural productivity through investments in infrastructure. Policies that foster behavioral change of the poor toward healthy diets are also needed. These actions will doubtlessly concrete the way to sustainable food security and nourishment, particularly for the underprivileged.

## Data Availability Statement

The raw data supporting the conclusions of this article will be made available by the authors, without undue reservation.

## Author Contributions

The conceptualization, formal analysis, and original draft is written by NA. The supervision and funding is provided by JH and data curation is done by AR. Writing, review, and editing is done by HS and WY. All authors contributed to the article and approved the submitted version.

## Conflict of Interest

The authors declare that the research was conducted in the absence of any commercial or financial relationships that could be construed as a potential conflict of interest.

## Publisher's Note

All claims expressed in this article are solely those of the authors and do not necessarily represent those of their affiliated organizations, or those of the publisher, the editors and the reviewers. Any product that may be evaluated in this article, or claim that may be made by its manufacturer, is not guaranteed or endorsed by the publisher.

## Footnotes

^1^The prevalence of undernourishment is the main variable used to measure hunger and is defined by Food and Agriculture Organization of the UN as the number of the population who has an insufficient caloric (dietary energy) intake to meet the minimum energy requirement.
